# Supplementary material for: Structural basis for molecular assembly of fucoxanthin chlorophyll a/c-binding proteins in a diatom photosystem I supercomplex
Source: eLife. 2024 Oct 31;13:RP99858. doi: 10.7554/eLife.99858 (PMC11527431; doi:10.7554/eLife.99858)
Supplement: Figure 1—figure supplement 1—source data 1. [file elife-99858-fig1-figsupp1-data1.zip › Figure 1-figure supplement 1-source data 1/Figure 1—figure supplement 1—source data 1.pdf]

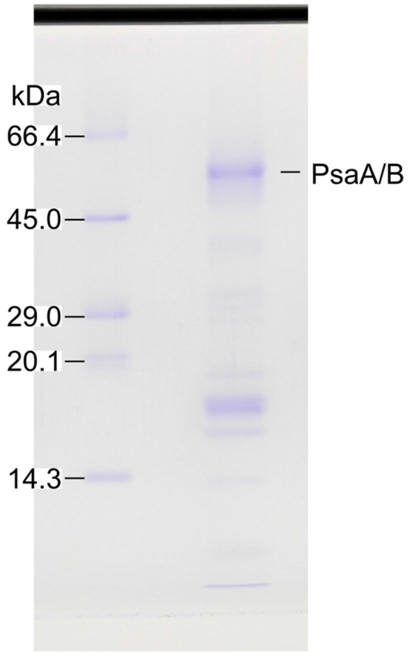

Figure 1—figure supplement 1—source data 1
Original file corresponding to Figure 1—figure supplement 1B.
